# Trimethyl 5-(2-chloro-4-fluoro­phen­yl)-2-phenyl­pyrrolidine-2,3,4-tricarboxyl­ate

**DOI:** 10.1107/S1600536809044274

**Published:** 2009-10-31

**Authors:** Long He, Lian-Mei Chen

**Affiliations:** a College of Chemistry and Chemical Engineering, China West Normal University, Nanchong 637002, People’s Republic of China

## Abstract

The title compound, C_22_H_21_ClFNO_6_, was synthesized by the 1,3-dipolar cyclo­addition reaction of dimethyl maleate, methyl 2-amino-2-phenyl­acetate and 2-chloro-4-fluoro­benzaldehyde. The pyrrolidine ring possesses an envelope conformation and the two benzene rings are oriented at a dihedral angle of 68.28 (7)°. Weak inter­molecular C—H⋯O hydrogen bonding is present in the crystal structure. One methyl group is disordered over two positions with a site-occupancy ratio of 0.651 (12):0.349 (12).

## Related literature

For the biological activity of pyrrolidine derivatives, see: Coldham & Hufton (2005 ▶); Nair & Suja (2007 ▶); Pandey *et al.* (2006 ▶); Sardina & Rapoport (1996 ▶); Witherup *et al.* (1995 ▶). For a related structure, see: Yu *et al.* (2007 ▶).
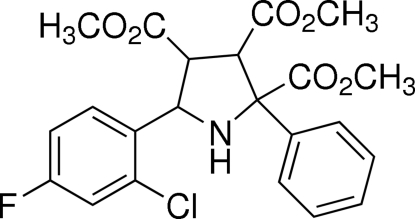


## Experimental

### 

#### Crystal data

C_22_H_21_ClFNO_6_
*M* *_r_* = 449.85
Orthorhombic, 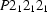

*a* = 9.474 (3) Å
*b* = 15.057 (8) Å
*c* = 15.182 (5) Å
*V* = 2165.7 (15) Å^3^
*Z* = 4
Cu *K*α radiation
μ = 1.98 mm^−1^
*T* = 298 K
0.38 × 0.36 × 0.03 mm

#### Data collection

Oxford Diffraction Gemini S Ultra diffractometer
Absorption correction: multi-scan (*CrysAlis Pro*; Oxford Diffraction, 2009 ▶) *T*_min_ = 0.520, *T*_max_ = 0.943
32943 measured reflections
3442 independent reflections
3332 reflections with *I* > 2σ(*I*)
*R*_int_ = 0.025

#### Refinement

*R*[*F* ^2^ > 2σ(*F* ^2^)] = 0.024
*wR*(*F* ^2^) = 0.060
*S* = 1.05
3442 reflections
290 parameters
1 restraint
H atoms treated by a mixture of independent and constrained refinement
Δρ_max_ = 0.12 e Å^−3^
Δρ_min_ = −0.16 e Å^−3^
Absolute structure: Flack (1983 ▶), 1456 Friedel pairs
Flack parameter: −0.001 (11)

### 

Data collection: *CrysAlis Pro* (Oxford Diffraction, 2009 ▶); cell refinement: *CrysAlis Pro*; data reduction: *CrysAlis Pro*; program(s) used to solve structure: *SHELXS97* (Sheldrick, 2008 ▶); program(s) used to refine structure: *SHELXL97* (Sheldrick, 2008 ▶); molecular graphics: *ORTEP-3* (Farrugia, 1997 ▶); software used to prepare material for publication: *SHELXL97*.

## Supplementary Material

Crystal structure: contains datablocks global, I. DOI: 10.1107/S1600536809044274/xu2655sup1.cif

Structure factors: contains datablocks I. DOI: 10.1107/S1600536809044274/xu2655Isup2.hkl

Additional supplementary materials:  crystallographic information; 3D view; checkCIF report

## Figures and Tables

**Table 1:** Hydrogen-bond geometry (Å, °)

*D*—H⋯*A*	*D*—H	H⋯*A*	*D*⋯*A*	*D*—H⋯*A*
C6—H6⋯O5^i^	0.93	2.56	3.380 (3)	147

Symmetry code: (i) 
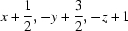
.

**Fig. 1.**
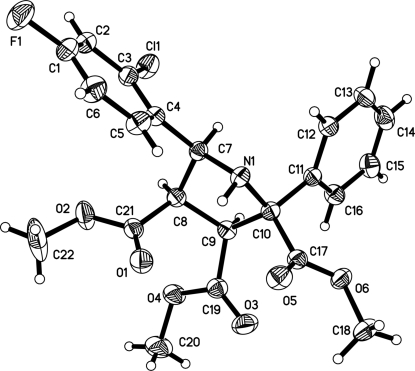
The molecular structure of (I) with 30% probability displacement ellipsoids (arbitrary spheres for H atoms).

## supplementary crystallographic information

### Comment

Substituted pyrrolidine compound is an important class of heterocyclic compounds with wide spread applications to the synthesis of biologically active compounds and natural products (Coldham *et al.*, 2005; Nair *et al.*, 2007; Pandey *et al.*, 2006; Sardina *et al.*, 1996; Witherup *et al.* 1995).

The molecular structure of (I) is shown in Fig. 1. Bond lengths and angles in (I) are normal. The pyrrolidine ring possesses an envelope conformation. The dihedral angle between the C1—C6 and C11—C16 benzene planes is 68.28 (7)°.

### Experimental

2-Chloro-4-fluorobenzaldehyde (0.063 g, 0.4 mmol), anhydrous sodium sulfate (200 mg) and dimethyl maleate (0.029 g, 0.2 mmol) were added to a solution of methyl 2-amino-2-phenylacetate(0.049 g, 0.3 mmol) in chloroform (2 ml). To the stirred mixture, acetic acid (0.012 g, 0.2 mmol) was added. After the mixture had been stirred at 323k for 10 h, the reaction was quenched with a saturated solution of sodium bicarbonate (5 ml). The mixture was extracted with diethyl ether, evaporated and separated by flash chromatograghy. A colourless powder was obtained. Single crystals suitable for X-ray diffraction were obtained by slow evaporation of an ethyl acetate solution.

### Refinement

Imino H atom was located in a difference Fourier map and positional parameters were refined, U_iso_(H) = 1.2U_eq_(N). The carbon-bound hydrogen atoms were placed in calculated positions, with C—H = 0.93–0.98 Å, and refined using a riding model, with U_iso_(H) =1.5U_eq_(C) for methyl H atoms and 1.2U_eq_(C) for the others. One of methyl groups is disordered over two positions, site occupancy factors were refined to 0.651 (12):0.349 (12).

### Crystal data

**Table:**

C_22_H_21_ClFNO_6_	*F*(000) = 936
*M**_r_* = 449.85	*D*_x_ = 1.380 Mg m^−^^3^
Orthorhombic, *P*2_1_2_1_2_1_	Cu *K*α radiation, λ = 1.54184 Å
Hall symbol: P 2ac 2ab	Cell parameters from 23516 reflections
*a* = 9.474 (3) Å	θ = 2.9–62.8°
*b* = 15.057 (8) Å	µ = 1.98 mm^−^^1^
*c* = 15.182 (5) Å	*T* = 298 K
*V* = 2165.7 (15) Å^3^	Platelet, colourless
*Z* = 4	0.38 × 0.36 × 0.03 mm

### Data collection

**Table:**

Oxford Diffraction Gemini S Ultra diffractometer	3442 independent reflections
Radiation source: Enhance Ultra (Cu) X-ray Source	3332 reflections with *I* > 2σ(*I*)
mirror	*R*_int_ = 0.025
Detector resolution: 15.9149 pixels mm^-1^	θ_max_ = 62.6°, θ_min_ = 4.1°
ω scans	*h* = −10→10
Absorption correction: multi-scan (*CrysAlis PRO*; Oxford Diffraction, 2009)	*k* = −17→17
*T*_min_ = 0.520, *T*_max_ = 0.943	*l* = −17→17
32943 measured reflections	

### Refinement

**Table:**

Refinement on *F*^2^	Hydrogen site location: inferred from neighbouring sites
Least-squares matrix: full	H atoms treated by a mixture of independent and constrained refinement
*R*[*F*^2^ > 2σ(*F*^2^)] = 0.024	*w* = 1/[σ^2^(*F*_o_^2^) + (0.0318*P*)^2^ + 0.3155*P*] where *P* = (*F*_o_^2^ + 2*F*_c_^2^)/3
*wR*(*F*^2^) = 0.060	(Δ/σ)_max_ = 0.001
*S* = 1.05	Δρ_max_ = 0.12 e Å^−^^3^
3442 reflections	Δρ_min_ = −0.16 e Å^−^^3^
290 parameters	Extinction correction: *SHELXL97* (Sheldrick, 2008), Fc^*^=kFc[1+0.001xFc^2^λ^3^/sin(2θ)]^-1/4^
1 restraint	Extinction coefficient: 0.0055 (2)
Primary atom site location: structure-invariant direct methods	Absolute structure: Flack (1983), 1456 Friedel pairs
Secondary atom site location: difference Fourier map	Flack parameter: −0.001 (11)

### Special details

**Table:**

Geometry. All e.s.d.'s (except the e.s.d. in the dihedral angle between two l.s. planes) are estimated using the full covariance matrix. The cell e.s.d.'s are taken into account individually in the estimation of e.s.d.'s in distances, angles and torsion angles; correlations between e.s.d.'s in cell parameters are only used when they are defined by crystal symmetry. An approximate (isotropic) treatment of cell e.s.d.'s is used for estimating e.s.d.'s involving l.s. planes.
Refinement. Refinement of *F*^2^ against ALL reflections. The weighted *R*-factor *wR* and goodness of fit *S* are based on *F*^2^, conventional *R*-factors *R* are based on *F*, with *F* set to zero for negative *F*^2^. The threshold expression of *F*^2^ > σ(*F*^2^) is used only for calculating *R*-factors(gt) *etc*. and is not relevant to the choice of reflections for refinement. *R*-factors based on *F*^2^ are statistically about twice as large as those based on *F*, and *R*- factors based on ALL data will be even larger.

### Fractional atomic coordinates and isotropic or equivalent isotropic displacement parameters (Å^2^)

**Table:**

	*x*	*y*	*z*	*U*_iso_*/*U*_eq_	Occ. (<1)
Cl1	0.87819 (6)	0.40571 (3)	0.69190 (3)	0.06039 (14)	
F1	1.10815 (14)	0.42498 (8)	0.39470 (7)	0.0789 (4)	
O1	0.64936 (15)	0.70388 (7)	0.55124 (8)	0.0578 (3)	
O2	0.59938 (18)	0.55992 (8)	0.53671 (8)	0.0715 (4)	
O3	0.50206 (14)	0.80786 (9)	0.73253 (11)	0.0735 (4)	
O4	0.41937 (11)	0.67622 (8)	0.68953 (9)	0.0568 (3)	
O5	0.79799 (13)	0.86655 (7)	0.67762 (8)	0.0553 (3)	
O6	0.74501 (13)	0.87248 (7)	0.82137 (7)	0.0509 (3)	
N1	0.89048 (14)	0.69866 (8)	0.69034 (9)	0.0390 (3)	
H1	0.870 (2)	0.7278 (11)	0.6438 (12)	0.047*	
C1	1.0498 (2)	0.46980 (13)	0.46390 (11)	0.0546 (4)	
C2	1.00115 (19)	0.42137 (12)	0.53368 (11)	0.0500 (4)	
H2	1.0094	0.3598	0.5350	0.060*	
C3	0.93922 (17)	0.46752 (10)	0.60228 (10)	0.0417 (4)	
C4	0.92532 (15)	0.55915 (10)	0.60251 (10)	0.0386 (3)	
C5	0.98023 (19)	0.60471 (12)	0.53049 (11)	0.0509 (4)	
H5	0.9745	0.6664	0.5293	0.061*	
C6	1.0431 (2)	0.56086 (13)	0.46054 (12)	0.0588 (5)	
H6	1.0796	0.5921	0.4128	0.071*	
C7	0.85178 (16)	0.60535 (9)	0.67778 (9)	0.0379 (3)	
H7	0.8783	0.5738	0.7318	0.045*	
C8	0.68738 (15)	0.60534 (10)	0.67347 (10)	0.0393 (3)	
H8	0.6491	0.5473	0.6902	0.047*	
C9	0.65018 (15)	0.67691 (10)	0.74326 (10)	0.0390 (3)	
H9	0.6296	0.6450	0.7980	0.047*	
C10	0.79423 (16)	0.72962 (10)	0.75935 (10)	0.0372 (3)	
C11	0.85468 (17)	0.70603 (9)	0.85036 (10)	0.0386 (3)	
C12	0.99477 (17)	0.68067 (11)	0.86012 (11)	0.0468 (4)	
H12	1.0537	0.6788	0.8111	0.056*	
C13	1.0475 (2)	0.65805 (12)	0.94231 (13)	0.0589 (5)	
H13	1.1410	0.6401	0.9477	0.071*	
C14	0.9633 (2)	0.66181 (12)	1.01602 (12)	0.0604 (5)	
H14	0.9992	0.6464	1.0710	0.073*	
C15	0.8256 (2)	0.68856 (12)	1.00731 (12)	0.0565 (5)	
H15	0.7685	0.6928	1.0569	0.068*	
C16	0.77124 (19)	0.70915 (11)	0.92558 (11)	0.0478 (4)	
H16	0.6769	0.7254	0.9207	0.057*	
C17	0.77865 (16)	0.83067 (10)	0.74725 (11)	0.0409 (3)	
C18	0.7123 (2)	0.96590 (11)	0.81177 (15)	0.0666 (5)	
H18A	0.7012	0.9922	0.8690	0.100*	
H18B	0.6263	0.9724	0.7790	0.100*	
H18C	0.7878	0.9950	0.7810	0.100*	
C19	0.51912 (18)	0.73007 (12)	0.72121 (11)	0.0462 (4)	
C20	0.2893 (2)	0.71857 (16)	0.66086 (19)	0.0810 (7)	
H20A	0.2229	0.6739	0.6429	0.121*	
H20B	0.3088	0.7573	0.6121	0.121*	
H20C	0.2502	0.7524	0.7086	0.121*	
C21	0.64215 (18)	0.63050 (10)	0.58152 (10)	0.0442 (4)	
C22A	0.5863 (10)	0.5705 (4)	0.4390 (3)	0.0813 (19)	0.651 (12)
H22A	0.6715	0.5505	0.4112	0.122*	0.651 (12)
H22B	0.5708	0.6319	0.4251	0.122*	0.651 (12)
H22C	0.5081	0.5358	0.4181	0.122*	0.651 (12)
C22B	0.5167 (16)	0.5848 (8)	0.4569 (7)	0.0813 (19)	0.349 (12)
H22D	0.4260	0.6070	0.4744	0.122*	0.349 (12)
H22E	0.5046	0.5335	0.4201	0.122*	0.349 (12)
H22F	0.5664	0.6300	0.4248	0.122*	0.349 (12)

### Atomic displacement parameters (Å^2^)

**Table:**

	*U*^11^	*U*^22^	*U*^33^	*U*^12^	*U*^13^	*U*^23^
Cl1	0.0909 (3)	0.0400 (2)	0.0503 (2)	0.0019 (2)	0.0084 (2)	0.00626 (17)
F1	0.0926 (9)	0.0837 (8)	0.0604 (6)	0.0170 (7)	0.0228 (6)	−0.0173 (6)
O1	0.0762 (9)	0.0468 (7)	0.0505 (7)	−0.0035 (6)	−0.0099 (6)	0.0073 (5)
O2	0.1081 (12)	0.0502 (7)	0.0562 (7)	−0.0174 (7)	−0.0325 (7)	0.0010 (6)
O3	0.0495 (7)	0.0544 (8)	0.1167 (13)	0.0096 (6)	−0.0117 (7)	−0.0141 (8)
O4	0.0371 (6)	0.0568 (7)	0.0766 (8)	−0.0006 (5)	−0.0088 (6)	−0.0026 (7)
O5	0.0692 (8)	0.0449 (6)	0.0519 (7)	0.0018 (5)	0.0049 (6)	0.0112 (5)
O6	0.0651 (8)	0.0368 (5)	0.0507 (6)	0.0065 (5)	−0.0016 (6)	−0.0044 (5)
N1	0.0423 (7)	0.0352 (6)	0.0393 (6)	−0.0019 (5)	0.0035 (6)	−0.0011 (5)
C1	0.0541 (10)	0.0627 (11)	0.0468 (10)	0.0104 (9)	0.0073 (8)	−0.0100 (8)
C2	0.0556 (10)	0.0442 (9)	0.0502 (9)	0.0081 (8)	−0.0030 (8)	−0.0062 (7)
C3	0.0452 (9)	0.0401 (8)	0.0399 (8)	0.0012 (7)	−0.0021 (7)	0.0014 (6)
C4	0.0372 (8)	0.0399 (8)	0.0387 (8)	0.0003 (6)	−0.0026 (6)	0.0005 (6)
C5	0.0582 (10)	0.0446 (9)	0.0499 (9)	0.0028 (8)	0.0104 (8)	0.0041 (7)
C6	0.0666 (12)	0.0620 (12)	0.0478 (10)	0.0016 (10)	0.0155 (9)	0.0046 (8)
C7	0.0412 (8)	0.0349 (7)	0.0376 (7)	−0.0011 (6)	0.0000 (6)	0.0004 (6)
C8	0.0409 (8)	0.0351 (7)	0.0419 (8)	−0.0020 (6)	−0.0008 (6)	0.0021 (6)
C9	0.0374 (8)	0.0407 (8)	0.0389 (8)	−0.0026 (7)	0.0007 (7)	0.0022 (6)
C10	0.0365 (8)	0.0372 (8)	0.0380 (8)	0.0002 (6)	0.0017 (6)	0.0001 (6)
C11	0.0420 (8)	0.0329 (7)	0.0410 (8)	−0.0010 (6)	−0.0030 (7)	0.0002 (6)
C12	0.0405 (9)	0.0459 (9)	0.0541 (10)	−0.0006 (7)	−0.0036 (7)	−0.0052 (7)
C13	0.0521 (11)	0.0548 (10)	0.0697 (12)	0.0039 (9)	−0.0210 (10)	0.0005 (9)
C14	0.0737 (14)	0.0547 (11)	0.0529 (11)	−0.0088 (10)	−0.0200 (10)	0.0087 (9)
C15	0.0655 (12)	0.0618 (10)	0.0421 (9)	−0.0092 (9)	−0.0007 (8)	0.0058 (8)
C16	0.0450 (9)	0.0541 (9)	0.0443 (9)	0.0005 (7)	0.0005 (7)	0.0030 (7)
C17	0.0386 (8)	0.0377 (8)	0.0465 (9)	−0.0006 (6)	−0.0021 (7)	0.0002 (7)
C18	0.0837 (13)	0.0395 (9)	0.0766 (13)	0.0132 (9)	−0.0060 (12)	−0.0070 (9)
C19	0.0404 (9)	0.0466 (10)	0.0517 (9)	0.0001 (7)	0.0018 (7)	−0.0010 (7)
C20	0.0436 (11)	0.0816 (15)	0.1177 (19)	0.0075 (10)	−0.0185 (12)	−0.0039 (14)
C21	0.0432 (9)	0.0443 (9)	0.0450 (8)	−0.0014 (7)	−0.0051 (7)	−0.0002 (7)
C22A	0.126 (6)	0.070 (2)	0.0474 (18)	−0.010 (3)	−0.027 (2)	−0.0089 (17)
C22B	0.126 (6)	0.070 (2)	0.0474 (18)	−0.010 (3)	−0.027 (2)	−0.0089 (17)

### Geometric parameters (Å, °)

**Table:**

Cl1—C3	1.7469 (16)	C8—H8	0.9800
F1—C1	1.366 (2)	C9—C19	1.515 (2)
O1—C21	1.1987 (19)	C9—C10	1.597 (2)
O2—C21	1.325 (2)	C9—H9	0.9800
O2—C22B	1.490 (7)	C10—C11	1.537 (2)
O2—C22A	1.497 (4)	C10—C17	1.540 (2)
O3—C19	1.195 (2)	C11—C12	1.389 (2)
O4—C19	1.335 (2)	C11—C16	1.390 (2)
O4—C20	1.454 (2)	C12—C13	1.387 (3)
O5—C17	1.201 (2)	C12—H12	0.9300
O6—C17	1.3283 (19)	C13—C14	1.375 (3)
O6—C18	1.448 (2)	C13—H13	0.9300
N1—C7	1.4645 (18)	C14—C15	1.371 (3)
N1—C10	1.465 (2)	C14—H14	0.9300
N1—H1	0.855 (18)	C15—C16	1.379 (2)
C1—C2	1.366 (3)	C15—H15	0.9300
C1—C6	1.373 (3)	C16—H16	0.9300
C2—C3	1.383 (2)	C18—H18A	0.9600
C2—H2	0.9300	C18—H18B	0.9600
C3—C4	1.386 (2)	C18—H18C	0.9600
C4—C5	1.392 (2)	C20—H20A	0.9600
C4—C7	1.508 (2)	C20—H20B	0.9600
C5—C6	1.385 (2)	C20—H20C	0.9600
C5—H5	0.9300	C22A—H22A	0.9600
C6—H6	0.9300	C22A—H22B	0.9600
C7—C8	1.559 (2)	C22A—H22C	0.9600
C7—H7	0.9800	C22B—H22D	0.9600
C8—C21	1.509 (2)	C22B—H22E	0.9600
C8—C9	1.552 (2)	C22B—H22F	0.9600
			
C21—O2—C22B	112.1 (5)	C12—C11—C10	121.02 (14)
C21—O2—C22A	116.7 (3)	C16—C11—C10	121.26 (14)
C22B—O2—C22A	28.9 (4)	C13—C12—C11	120.50 (17)
C19—O4—C20	116.19 (15)	C13—C12—H12	119.7
C17—O6—C18	115.25 (14)	C11—C12—H12	119.7
C7—N1—C10	104.04 (11)	C14—C13—C12	120.89 (17)
C7—N1—H1	109.1 (12)	C14—C13—H13	119.6
C10—N1—H1	106.6 (12)	C12—C13—H13	119.6
F1—C1—C2	117.99 (17)	C15—C14—C13	119.02 (17)
F1—C1—C6	118.91 (17)	C15—C14—H14	120.5
C2—C1—C6	123.10 (16)	C13—C14—H14	120.5
C1—C2—C3	117.32 (16)	C14—C15—C16	120.55 (18)
C1—C2—H2	121.3	C14—C15—H15	119.7
C3—C2—H2	121.3	C16—C15—H15	119.7
C2—C3—C4	122.85 (15)	C15—C16—C11	121.28 (17)
C2—C3—Cl1	117.35 (12)	C15—C16—H16	119.4
C4—C3—Cl1	119.79 (12)	C11—C16—H16	119.4
C3—C4—C5	116.95 (14)	O5—C17—O6	124.68 (14)
C3—C4—C7	120.33 (13)	O5—C17—C10	122.33 (14)
C5—C4—C7	122.72 (14)	O6—C17—C10	112.97 (13)
C6—C5—C4	121.88 (16)	O6—C18—H18A	109.5
C6—C5—H5	119.1	O6—C18—H18B	109.5
C4—C5—H5	119.1	H18A—C18—H18B	109.5
C1—C6—C5	117.86 (17)	O6—C18—H18C	109.5
C1—C6—H6	121.1	H18A—C18—H18C	109.5
C5—C6—H6	121.1	H18B—C18—H18C	109.5
N1—C7—C4	115.18 (12)	O3—C19—O4	123.47 (16)
N1—C7—C8	104.82 (12)	O3—C19—C9	126.67 (16)
C4—C7—C8	115.45 (12)	O4—C19—C9	109.81 (14)
N1—C7—H7	107.0	O4—C20—H20A	109.5
C4—C7—H7	107.0	O4—C20—H20B	109.5
C8—C7—H7	107.0	H20A—C20—H20B	109.5
C21—C8—C9	113.13 (12)	O4—C20—H20C	109.5
C21—C8—C7	108.82 (12)	H20A—C20—H20C	109.5
C9—C8—C7	101.43 (11)	H20B—C20—H20C	109.5
C21—C8—H8	111.0	O1—C21—O2	124.03 (15)
C9—C8—H8	111.0	O1—C21—C8	124.77 (14)
C7—C8—H8	111.0	O2—C21—C8	111.13 (13)
C19—C9—C8	113.72 (13)	O2—C22A—H22A	109.5
C19—C9—C10	118.14 (13)	O2—C22A—H22B	109.5
C8—C9—C10	104.80 (12)	H22A—C22A—H22B	109.5
C19—C9—H9	106.5	O2—C22A—H22C	109.5
C8—C9—H9	106.5	H22A—C22A—H22C	109.5
C10—C9—H9	106.5	H22B—C22A—H22C	109.5
N1—C10—C11	109.71 (12)	O2—C22B—H22D	109.5
N1—C10—C17	106.78 (12)	O2—C22B—H22E	109.5
C11—C10—C17	111.80 (12)	H22D—C22B—H22E	109.5
N1—C10—C9	105.33 (12)	O2—C22B—H22F	109.5
C11—C10—C9	109.94 (12)	H22D—C22B—H22F	109.5
C17—C10—C9	113.00 (12)	H22E—C22B—H22F	109.5
C12—C11—C16	117.72 (15)		
			
F1—C1—C2—C3	−178.45 (16)	N1—C10—C11—C12	14.85 (19)
C6—C1—C2—C3	1.8 (3)	C17—C10—C11—C12	−103.41 (16)
C1—C2—C3—C4	0.0 (3)	C9—C10—C11—C12	130.23 (15)
C1—C2—C3—Cl1	−179.53 (14)	N1—C10—C11—C16	−165.02 (14)
C2—C3—C4—C5	−1.5 (2)	C17—C10—C11—C16	76.71 (18)
Cl1—C3—C4—C5	177.95 (12)	C9—C10—C11—C16	−49.64 (18)
C2—C3—C4—C7	177.68 (14)	C16—C11—C12—C13	1.0 (2)
Cl1—C3—C4—C7	−2.8 (2)	C10—C11—C12—C13	−178.87 (15)
C3—C4—C5—C6	1.5 (3)	C11—C12—C13—C14	−1.2 (3)
C7—C4—C5—C6	−177.73 (16)	C12—C13—C14—C15	−0.1 (3)
F1—C1—C6—C5	178.39 (17)	C13—C14—C15—C16	1.7 (3)
C2—C1—C6—C5	−1.9 (3)	C14—C15—C16—C11	−1.9 (3)
C4—C5—C6—C1	0.2 (3)	C12—C11—C16—C15	0.6 (2)
C10—N1—C7—C4	173.79 (12)	C10—C11—C16—C15	−179.57 (15)
C10—N1—C7—C8	45.80 (14)	C18—O6—C17—O5	8.4 (2)
C3—C4—C7—N1	156.57 (14)	C18—O6—C17—C10	−172.84 (15)
C5—C4—C7—N1	−24.3 (2)	N1—C10—C17—O5	24.6 (2)
C3—C4—C7—C8	−80.97 (18)	C11—C10—C17—O5	144.60 (15)
C5—C4—C7—C8	98.19 (18)	C9—C10—C17—O5	−90.74 (18)
N1—C7—C8—C21	81.72 (14)	N1—C10—C17—O6	−154.16 (13)
C4—C7—C8—C21	−46.10 (17)	C11—C10—C17—O6	−34.15 (18)
N1—C7—C8—C9	−37.78 (14)	C9—C10—C17—O6	90.51 (15)
C4—C7—C8—C9	−165.60 (12)	C20—O4—C19—O3	5.6 (3)
C21—C8—C9—C19	30.15 (18)	C20—O4—C19—C9	−176.82 (16)
C7—C8—C9—C19	146.52 (13)	C8—C9—C19—O3	−140.85 (18)
C21—C8—C9—C10	−100.31 (14)	C10—C9—C19—O3	−17.4 (3)
C7—C8—C9—C10	16.07 (14)	C8—C9—C19—O4	41.67 (18)
C7—N1—C10—C11	84.15 (14)	C10—C9—C19—O4	165.13 (13)
C7—N1—C10—C17	−154.52 (12)	C22B—O2—C21—O1	−19.4 (7)
C7—N1—C10—C9	−34.13 (14)	C22A—O2—C21—O1	12.0 (5)
C19—C9—C10—N1	−117.73 (14)	C22B—O2—C21—C8	163.6 (7)
C8—C9—C10—N1	10.09 (15)	C22A—O2—C21—C8	−165.1 (5)
C19—C9—C10—C11	124.14 (14)	C9—C8—C21—O1	38.7 (2)
C8—C9—C10—C11	−108.04 (13)	C7—C8—C21—O1	−73.2 (2)
C19—C9—C10—C17	−1.53 (19)	C9—C8—C21—O2	−144.27 (15)
C8—C9—C10—C17	126.29 (13)	C7—C8—C21—O2	103.82 (16)

### Hydrogen-bond geometry (Å, °)

**Table:**

*D*—H···*A*	*D*—H	H···*A*	*D*···*A*	*D*—H···*A*
C6—H6···O5^i^	0.93	2.56	3.380 (3)	147

Symmetry codes: (i) *x*+1/2, −*y*+3/2, −*z*+1.
